# Identification of Metabolic Pathways Influenced by the G-Protein Coupled Receptors GprB and GprD in *Aspergillus nidulans*

**DOI:** 10.1371/journal.pone.0062088

**Published:** 2013-05-01

**Authors:** Wagner R. de Souza, Enyara Rezende Morais, Nadia Graciele Krohn, Marcela Savoldi, Maria Helena S. Goldman, Fernando Rodrigues, Camila Caldana, Charles T. Semelka, Andrey P. Tikunov, Jeffrey M. Macdonald, Gustavo Henrique Goldman

**Affiliations:** 1 Laboratório Nacional de Ciência e Tecnologia do Bioetanol-CTBE, Campinas, São Paulo, Brazil; 2 Faculdade de Ciências Farmacêuticas de Ribeirão Preto, Universidade de São Paulo, Ribeirão Preto, São Paulo, Brazil; 3 Faculdade de Filosofia, Ciências e Letras de Ribeirão Preto, Universidade de São Paulo, Ribeirão Preto, São Paulo, Brazil; 4 Life and Health Sciences Research Institute (ICVS), School of Health Sciences, University of Minho, Campus de Gualtar, Braga, Portugal; 5 Department of Biomedical Engineering and UNC Metabolomic Facility, University of North Carolina, Chapel Hill, North Carolina, United States of America; University of Medicine & Dentistry of New Jersey - New Jersey Medical School, United States of America

## Abstract

Heterotrimeric G-protein-mediated signaling pathways play a pivotal role in transmembrane signaling in eukaryotes. Our main aim was to identify signaling pathways regulated by *A. nidulans* GprB and GprD G-protein coupled receptors (GPCRs). When these two null mutant strains were compared to the wild-type strain, the *ΔgprB* mutant showed an increased protein kinase A (PKA) activity while growing in glucose 1% and during starvation. In contrast, the *ΔgprD* has a much lower PKA activity upon starvation. Transcriptomics and ^1^H NMR-based metabolomics were performed on two single null mutants grown on glucose. We noted modulation in the expression of 11 secondary metabolism gene clusters when the *ΔgprB* and *ΔgprD* mutant strains were grown in 1% glucose. Several members of the sterigmatocystin-aflatoxin gene cluster presented down-regulation in both mutant strains. The genes of the NR-PKS monodictyphenone biosynthesis cluster had overall increased mRNA accumulation in *ΔgprB*, while in the *ΔgprD* mutant strain the genes had decreased mRNA accumulation. Principal component analysis of the metabolomic data demonstrated that there was a significant metabolite shift in the *ΔgprD* strain. The ^1^H NMR analysis revealed significant expression of essential amino acids with elevated levels in the *ΔgprD* strain, compared to the wild-type and *ΔgprB* strains. With the results, we demonstrated the differential expression of a variety of genes related mainly to secondary metabolism, sexual development, stress signaling, and amino acid metabolism. We propose that the absence of GPCRs triggered stress responses at the genetic level. The data suggested an intimate relationship among different G-protein coupled receptors, fine-tune regulation of secondary and amino acid metabolisms, and fungal development.

## Introduction

Signal transduction pathways are essential for the living organisms, controlling the majority of physiological responses. Heterotrimeric G-protein-mediated signaling pathways play a pivotal role in transmembrane signaling in eukaryotes, through the modulation of an extracellular stimulus and its transmission within the cell. G-protein signaling is comprised of three parts: (i) a seven-transmembrane-spanning G-protein coupled receptor (GPCR); (ii) a heterotrimeric G protein consisting of α, β and γ subunits, and (iii) an effector. After sensitization of a specific GPCR by ligand binding, conformational changes of the Gα subunit occur, resulting in an exchange of GDP to GTP in this subunit. The GTP-bound Gα subunit undergoes another conformational switch, promoting the dissociation of Gα-GTP and Gβγ subunits, and both separated subunits can amplify and propagate signals by modulating activities of effector molecules [1].

Fungi are excellent models to study environmental sensing because they have simple but evolutionarily conserved signal transduction pathways similar to those present in many eukaryotes. Microorganisms, such as fungi, have evolved mechanisms in order to sense and adapt to their environment. In this context, GPCRs are the largest family of transmembrane receptors that are able to sense signals, especially pheromones and nutrients [2]. The pathways regulated through GPCRs control various aspects of growth, morphogenesis, mating, and virulence [3]. A combinatorial search of various fungal genomes was performed in order to identify putative GPCRs in the model filamentous fungus *Aspergillus nidulans*. The results identified nine genes predicted to encode GPCRs, designated GprA to GprI, and subsequent phylogenetic analyses grouped them into four classes [4]. Subsequent studies on *A. nidulans* demonstrated that seventeen geens, designated GprA-P and NopA, actually encode for at least sixteen putative GPCRs [5], [6].

GprB and GprD were previously characterized in *A. nidulans* [4], [7]. GprB is similar to the pheromone receptor Ste3p of *S. cerevisiae* [4] and appears to be required for self-fertilization in *A. nidulans*. A null *gprB* mutant affects self-fertilized fruiting body formation (without affecting vegetative growth), asexual development, Hülle cell production and heterothallic sexual development [7]. GprD is required to negatively modulate sexual development in *A. nidulans* based on results showing a null *gprD* mutant led to uncontrolled activation of sexual development, and subsequent developmental abnormalities [4]. All the promoter regions of *gprB/D* contain two or three copies of a pentanucleotide stress response element (STRE; 5′-AGGGG-3′), and various stresses can affect sexual development in fungi [7]. A recent study proposed that the ligand for GprD belongs to a class of hormones known as oxylipins [8]. Treatment of *A. nidulans* wild-type with plant oxylipins results in cAMP accumulation, but this is prevented in the absence of the *gprD* gene. Despite the recent characterization of GprB and GprD, the signals and mechanisms responsible for their functions remain to be characterized. Therefore, we identified signaling pathways regulated by *A. nidulans* GprB and GprD. Transcriptomics of two single mutants grown on glucose was performed to quantify the differential gene expression in the strains lacking *gprB* and *gprD*, compared to the wild-type strain. In addition, metabolomic analysis using ^1^H nuclear magnetic resonance (NMR) spectroscopy generated a metabolic fingerprint comparing levels of many compounds present in the wild-type and the mutant strains grown in glucose. Our results present a broad view of metabolic pathways affected by GprB and GprD in *A. nidulans*.

## Results and Discussion

### Phenotypical characterization of A. nidulans strains grown on different carbon sources

**Figure 1:**
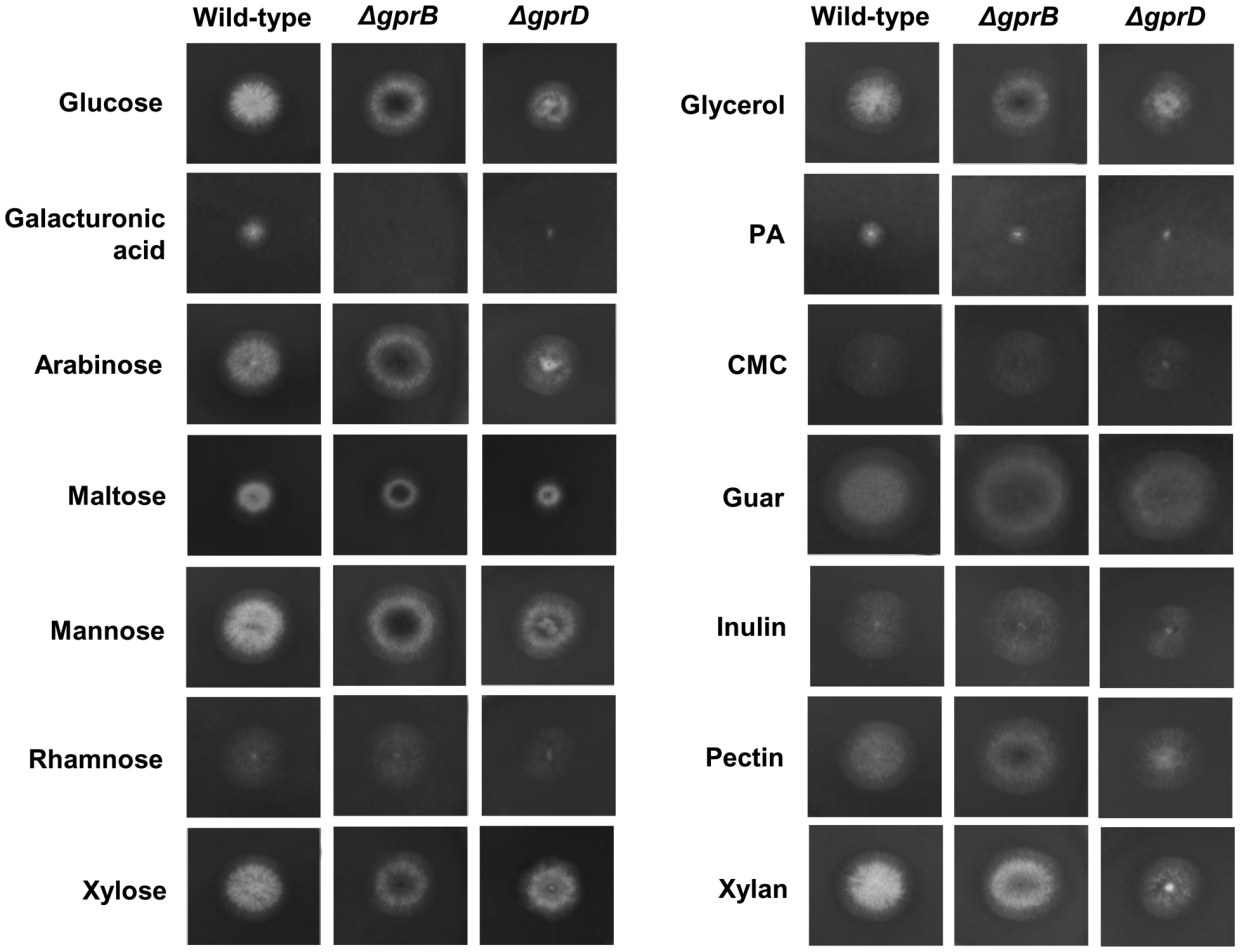
The *ΔgprB* and *ΔgprD* growth on different carbon sources. Growth phenotypes of strains having the indicated relevant, partial genotypes on the indicated solid media after 72 hours at 37°C.

In a first attempt to elucidate the biological role of GprD and the putative pheromone receptor GprB in *A. nidulans*, corresponding gene deletion mutants were grown on different carbon sources to validate that the absence of *gprB* or *gprD* genes affects carbon utilization. The growth of the *ΔgprB*, *ΔgprD*, and the wild-type strains was analyzed on minimal medium (MM) containing mono-(25 mM each) and polysaccharydes (1% each) and glycerol (2%) grown at 37°C. As shown in Figure 1, under all tested conditions, the radial diameter of the colonies of the mutant strains was comparable to that of the wild-type strain. Similarly, there were no growth differences at 30, 37, and 44°C (data not shown). These results indicate that the mutant strains are able to metabolize all tested sugars. Therefore, the corresponding deleted genes are not essential sensor molecules for growth on the carbon sources tested.

**Figure 2:**
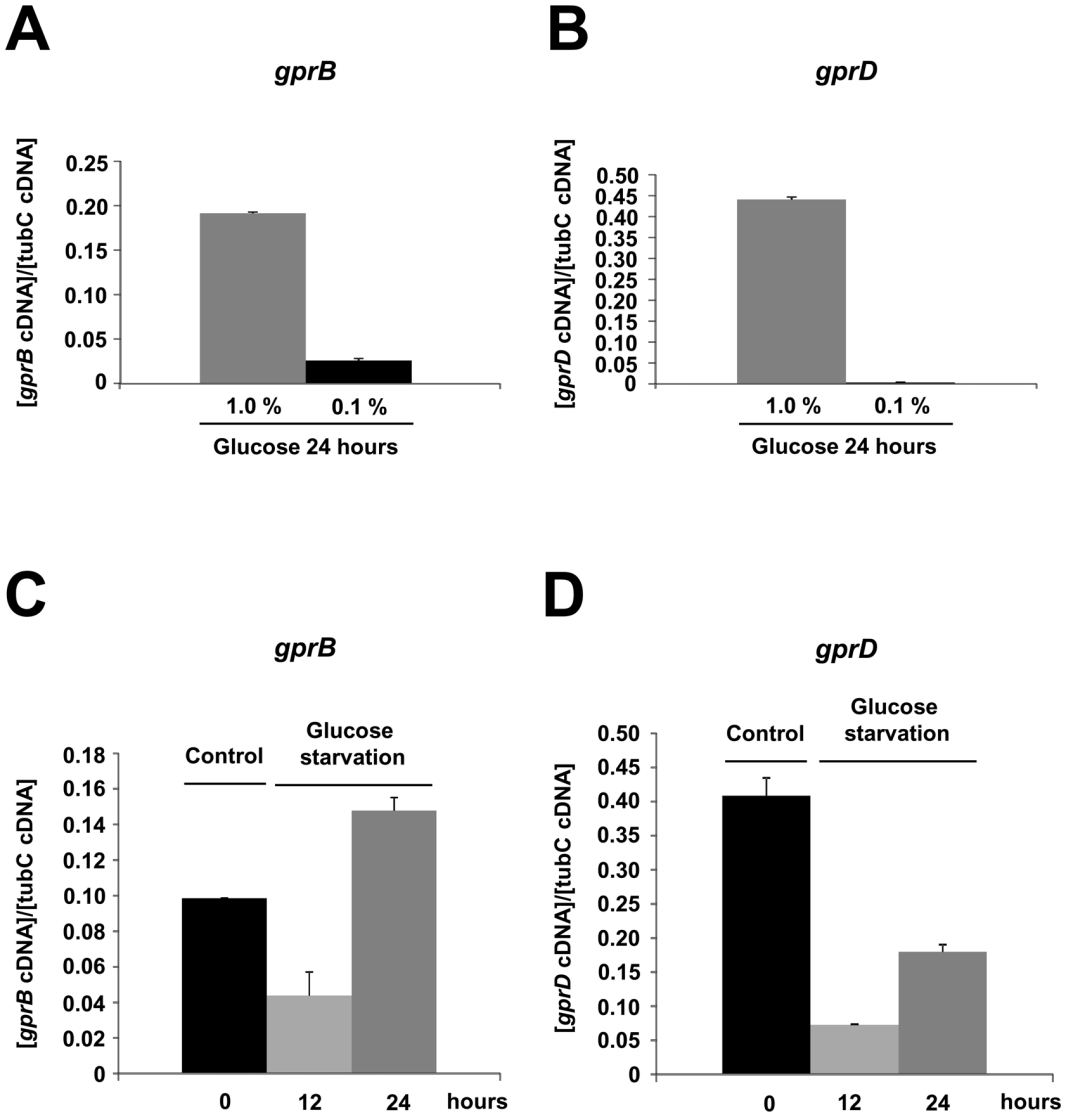
The *gprB* and–*D* mRNA accumulation levels in different growth conditions in the wild-type strain. (A) and (C) gprB; (B) and (D) *gprD*.

The accumulation of mRNA encoding for GprB and GprD was quantified in wild-type at different glucose concentrations and during carbon starvation (Figure 2). There was an increase in *gprB* and *gprD* mRNA accumulation when the wild-type strain was grown in 1% glucose (Figures 2A–B), while only the *gprB* mRNA accumulated at the lower glucose level of 0.1% (Figure 2A). We also evaluated the mRNA accumulation of *gprB* and *gprD* during carbon starvation in wild-type (Figures 2C–D). Both genes had a decrease in their mRNA accumulation after 12 hours of starvation (Figures 2C–D); however at 24 hours of starvation, there is an increase in *gprB* and a decrease in *gprD* mRNA accumulation (Figures 2C–D). Curiously, the *gprB* mRNA levels were comparable after 24 hours of starvation and when they were grown in 1% glucose (Figure 2). We cannot currently provide a reasonable explanation for this observation.

**Figure 3:**
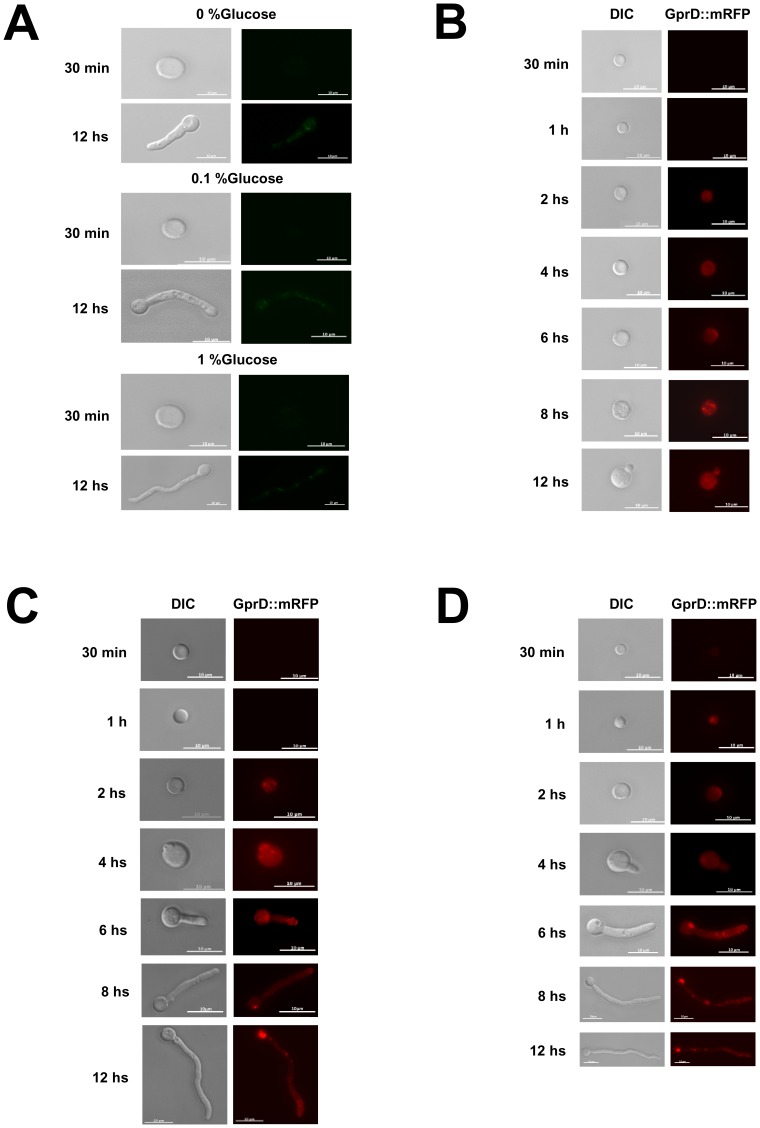
Sub-cellular location of GprB and–D. (A) GprB::GFP, (B) GprD::mRFP (grown in MM+0% glucose), (C) GprD::mRFP (grown in MM+0.1% glucose), and (D) GprD::mRFP (grown in MM+1.0% glucose) at 30°C (Bars, 10 µm).

We constructed GprB::GFP and GprD::mRFP strains aiming to verify their sub-cellular localization. These two strains have identical phenotypes to the wild-type strain (data not shown). In general, receptors located in the cellular membrane need to be desensitized after activation by their ligands in order to avoid a constitutive signal. In this way, receptors are internalized and can be either degraded or recycled [9], [10]. We have followed their sub-cellular location by germinating them for 30 minutes, 1, 2, 4, 6, 8, and 12 hours at 37°C in either MM without any carbon source, 0.1% glucose or 1.0% glucose. In the earlier time points like 30 minutes and 1, 2, 4, and 8 hours the GprB::GFP was not visible (Figure 3A and data not shown). However at 12 hours germination in all media, this fluorescence appeared diffused in the cytoplasm (Figure 3A). In contrast, GprD::mRFP showed a detectable fluorescence for all time points and media (Figure 3B). When conidia were germinated in MM without any carbon source, there is fluorescence at 2 to 12 hours localized more intensely at the periphery of the swollen conidia, probably concentrated at the emerging apical tip of future germling (Figure 3B). In MM+0.1% glucose, GprD::mRFP is present at the periphery of the germling at 2, 4, 6, and 8 hours, starts to be removed to the single vacuole (12 hours; Figure 3C; second lane). In MM+1.0% glucose, there is a strong accumulation of GprD::mRFP at the periphery of the cell at 30 minutes, 1, 2, and 4 hours (Figure 3D). From 6 to 12 hours, there is intense formation of vesicles and translocation to the single vacuole (Figure 3D).

**Figure 4:**
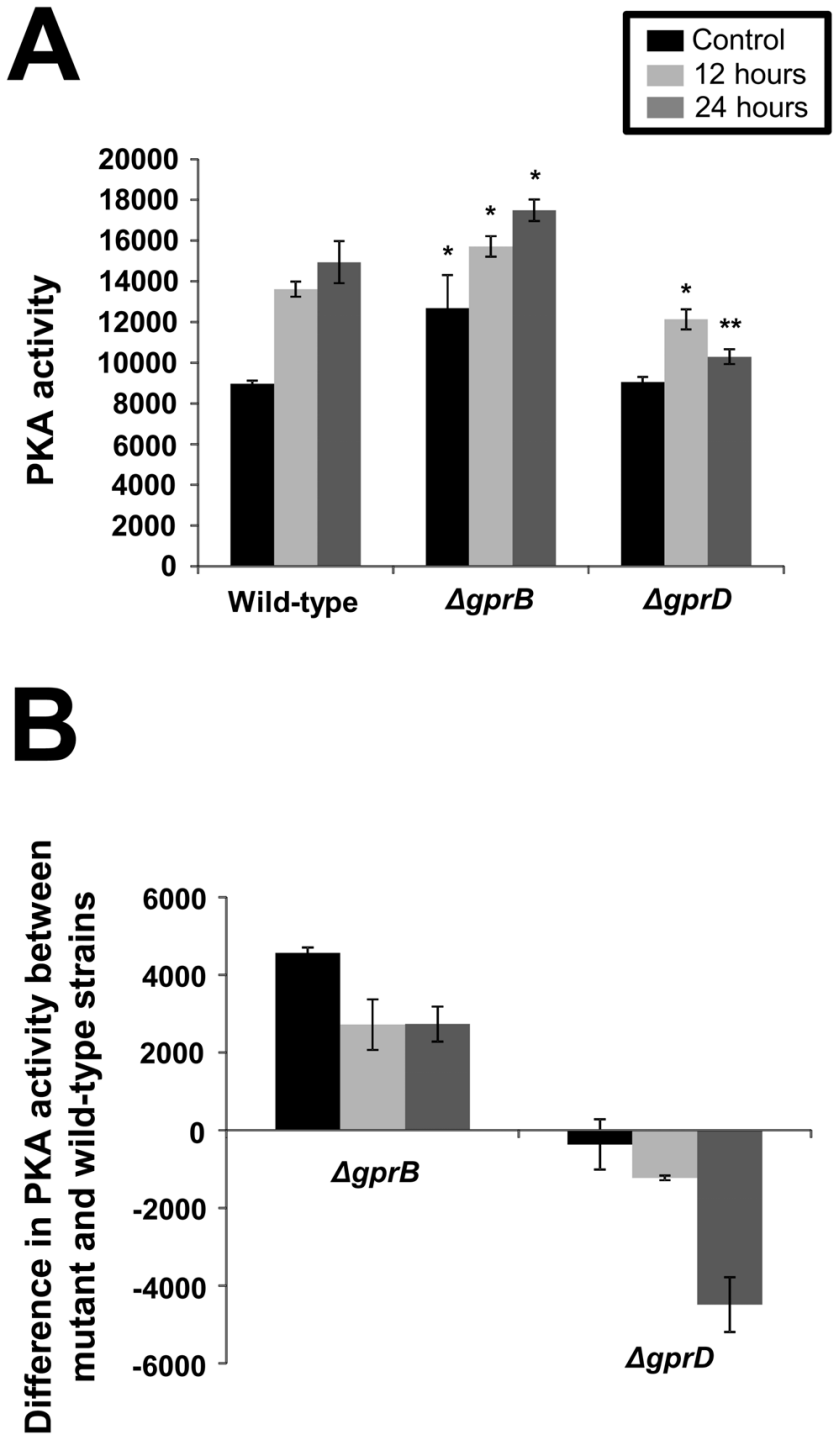
Determination of the protein kinase A activity for the wild-type, *ΔgprB* and *ΔgprD* mutant strains. Protein kinase A (PKA) activity is increased and decreased in the *ΔgprB* and *ΔgprD* mutant strains, respectively, upon carbon starvation. These three strains were grown for 24 hours in liquid minimal medium supplemented with 2% glucose. Then, their mycelia were transferred to liquid minimal medium without carbon source for 12 and 24 hours. The control represents PKA activity before transferring the mycelia to liquid medium without any carbon source. (A) Absolute levels of PKA activity from cultures of the wild-type, *ΔgprB*, and *ΔgprD*mutant strains for control MM+2% glucose) and carbon-starved for 12 and 24 hours. (B) Difference in PKA activity between the wild-type strain and *ΔgprB* and *ΔgprD*mutant strains. One unit of kinase activity is defined as the number of nanomoles of phosphate transferred to a substrate per minute per milliliter. The *t*-test was used to compare the mutant strains with the wild-type strain (*p*-value<0.05, **, and<0.01 *).

Protein kinase A (PKA) has a very important role in growth. PKA helps cells to respond to glucose and links cell cycle progression to mass accumulation (for a review, see [11], [12]). *S. cerevisiae* Gpa2, a Gα subunit of the heterotrimeric G proteins, and Gpr1, a G protein-coupled receptor, that physically interacts with Gpa2, define a nutrient-sensing pathway that works in parallel with Ras to activate PKA (for a review, see [12]). Several studies suggest that Gpr1/Gpa2 activate PKA through activation of adenylate cyclase [13], [14]. We evaluated how starvation affects the PKA activity in the wild-type, *ΔgprB*, and *ΔgprD* mutant strains. By growing them for 24 hours in liquid minimal medium supplemented with 2% glucose, and then tranferring the mycelia to a liquid minimal medium without a carbon source for 12 and 24 hours (Figure 4). Upon starvation, all three strains displayed increased PKA activity (Figure 4). When these two null mutants were compared to the wild-type strain, the *ΔgprB* mutant showed an increased PKA activity in both the control (before transferring to liquid medium without any carbon source) and during starvation (Figure 4A). In contrast, the *ΔgprD* had a much lower PKA activity upon starvation (Figure 4B).

During *S. cerevisiae* growth, high PKA activity occurs in the presence of rapidly fermented sugars like glucose or sucrose. Growth arrest of fermenting cells or growth on a respiratory carbon source, like glycerol or ethanol, is associated with low activity of the PKA pathway. *S. cerevisiae* PKA is inactive, existing as a tetrameric holoenzyme composed of two catalytic subunits encoded by one of three redundant *TPK* genes (*TPK1*, *TPK2*, and *TPK3*) and two regulatory subunits encoded by *BCY1* [11], [12], [15]. The addition of glucose to cells induces a rapid elevation of the cAMP level due to activation of adenylate cyclase (Cyr1) via the Gpr1/Gpa2 and the Ras1/Ras2 pathways [16]. Binding of cAMP to the Bcy1 inhibitory subunit of PKA liberates the catalytic subunits, resulting in their activation [16]. Glucose-starvation for 12 and 24 hours in *A. nidulans* induced a 60% increase in PKA activity. Interestingly, *ΔgprB* and *ΔgprD* showed contrasting behavior, *i.e.* the lack of GprB promoted an increase in PKA activity during growth on glucose and during starvation. On the other hand, the absence of GprD greatly reduced PKA activity under starvation conditions.

### Transcriptome analysis of A. nidulans ΔgprB and ΔgprB strains grown on glucose

**Figure 5:**
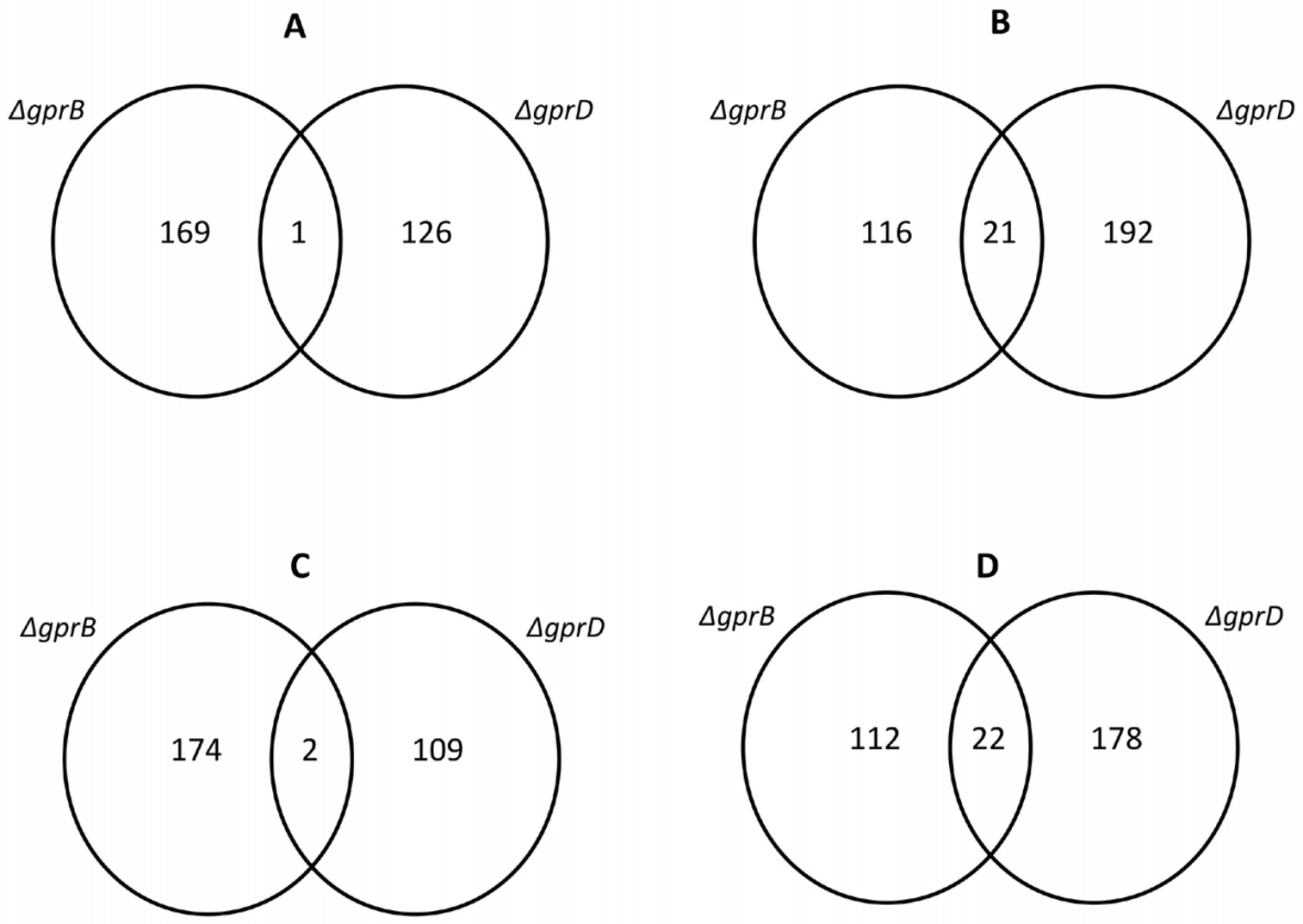
Venn diagram showing the genes specifically expressed in the *ΔgprB* and *ΔgprD* mutant strains. (A) and (B) show the genes up-regulated at 24 and 48 hours, respectively, while (C) and (D) show the genes down-regulated at 24 and 48 hours, respectively.

To gain insight into which genes were influenced by the absence of *gprB* or *gprD* genes, we determined the transcriptional profile of *A. nidulans* strains grown on glucose 1%. The wild-type, *ΔgprB*, and *ΔgprD* strains were grown on 1% glucose for 24 and 48 hours at 37°C. The wild-type strain was used as reference, and differentially expressed genes were observed in the mutant strains. Only significant (p<0.01) differentially expressed genes, up-or down-regulated, were considered with log_2_ Cy5/Cy3 ratios≥1 and≤1. In the *ΔgprB* strain we observed 308 and 311 differentially expressed genes at 24 and 48 hours, respectively, and 341 and 312 genes in the *ΔgprD* strain at 24 and 48 hours, respectively (Supplementary Table S1). The Venn diagram for the differentially expressed genes (Figure 5) demonstrated that 1 gene was up-regulated in both mutant strains grown on glucose for 24 hours (Figure 5A), and 21 genes were down-regulated in the same conditions (Figure 5B). Upon 48 hours of growth, 2 genes were up-regulated (Figure 5C), and 22 genes were down-regulated in both mutant strains (Figure 5D). Included in the 21 genes down-regulated at 24 hours were seven genes that encode proteins involved with sterigmatocystin biosynthesis and AN10576, encoding *ivoA*, one module nonribosomal peptide synthetase (NRPS) involved in conidiophore pigmentation ([17]; Tables S1 and S2).

Taken together, the results suggest that the absence of *gprB* and *gprD* genes interfered with different pathways of *A. nidulans* at the transcriptional level. FunCat, a genetic database, functionally categorized the differentially expressed genes with known classification (Functional Catalogue database, http://mips.helmholtz-muenchen.de/proj/funcatDB) (Table 1, Supplementary Table S2). The majority of differentially transcribed genes in the mutant strains respond to the category "Metabolism" and the subcategories "Carbohydrate and Secondary metabolism". The transcriptome analysis reveals that the absence of *gprB* and *gprD* genes interferes with overall metabolism, due to disturbances in carbon flow, and these effects were more pronounced in the *ΔgprD* strain. After growth of *ΔgprD* strain on glucose, three genes encoding key metabolic enzymes involved in primary metabolism were strongly down-regulated: AN2583 (*gpdC*), encoding glyceraldehyde-3-phosphate dehydrogenase (GAPDH), AN5843 (*pdkA*), encoding phosphoenolpyruvate synthase (PEP synthase), and AN4913 (*phk*), encoding phosphoketolase (PHK; Supplementary Table S1). The GAPDH plays a central role in glycolysis, where it converts glyceraldehyde-3-phosphate to bisphosphoglycerate. PEP synthase catalyzes the first step of conversion of pyruvate to glucose in gluconeogenesis. PHK plays a pivotal role in the pentose phosphate pathway (PPP), converting D-xylulose-5-phosphate into acetyl phosphate and glyceraldehyde-3-phosphate. The three genes, cited above, were less expressed by 3.5-fold in the *ΔgprD* grown on glucose, compared to the wild-type strain. The results demonstrate disturbances in the carbon flow of glucose metabolism in *ΔgprD*. In contrast, the gene encoding an ATP-citrate lyase (ACL; AN 3577) was more expressed by 2-fold in the *ΔgprD* strain compared to the wild-type. ACL is an enzyme responsible for providing cytoplasmic acetyl-CoA from citrate [18]. In a previous study, ACL was required for normal asexual and sexual development in the ascomycete fungus *Fusarium graminearum* [19].

**Table 1:** Categorization of genes differentially expressed in *A. nidulans* mutant strains grown on glucose compared to the wild-type strain.

FunCat category*	▵*gprB*(down-regulated)	▵*gprB*(up-regulated)	▵*gprD*(down-regulated)	▵*gprD*(up-regulated)
Metabolism	27	39	43	16
Cell rescue, defense and virulence	5	10	8	9
Transport	9	7	11	15
Transcription	1	1	2	3
Protein synthesis	1	-	-	
Protein fate	-	1	4	1
Protein with binding functions	1	7	9	5
Biogenesis of cellular components	2	1	1	
Cell type differentiation	1	1	1	
Signal transduction	-	1	1	1
Interaction with the environment	-	5	1	3
Energy	-	-	2	1
Regulation of metabolism	-	-	-	1
Retroelements	-	-	-	2
Biogenesis	-	-	-	2

* Categorization enrichment analysis were performed with p<0.001.

G-protein signaling is strictly connected to secondary metabolite production and fungal development [20]–[25]. A study estimated 53 secondary metabolism gene clusters encode for backbone structures in *A. nidulans*, such as putative polyketide synthase (PKS), non-ribosomal peptide synthase (NRPS), and hybrid PKS-NRPS genes [26]. The results of this study show modulated expression of 11 secondary metabolism gene clusters encoding for backbone structures when the *ΔgprB* and *ΔgprD* mutant strains are grown in 1% glucose for 24 and 48 hours (Suplementary Tables S1 and S2). Several members of the sterigmatocystin-aflatoxin gene cluster presented down-regulation in both mutant strains (AN7806, AN7807, AN7812, AN7816, AN7818, AN7825, and AN11013). Notably, most of the genes of the NR-PKS monodictyphenone biosynthesis cluster are differentially regulated in both mutants (Supplementary Tables S1 and S2). In *ΔgprB*, the NR-PKS genes have increased mRNA accumulation. In the *ΔgprD* strain they have decreased mRNA accumulation (Supplementary Tables S1 and S2).

Three genes encoding oxidoreductases similar to sporulation-related genes in *S. cerevisiae* were up-regulated in *ΔgprB* (AN9127, AN0216, and AN2606). In the *ΔgprD* strain, the gene encoded by AN2606 was strongly down-regulated (6-fold difference compared to the wild-type) (Supplementary Table S1). The gene AN2606 is similar to *S. cerevisiae* gene *dit1*, a gene encoding for a pyoverdine/dityrosine biosynthesis protein involved in spore cell wall maturation [27]. Moreover, in the *ΔgprD* strain, two genes encoding proteins related to conidium development and sporulation were strongly deregulated, AN8803 and AN0055. AN8803 encodes RodA, a putative hydrophobin transcriptionally regulated by BrlA, a transcription factor required for normal sporulation in *Aspergilli* [28], [29]. AN0055 encodes for TmpA, a putative transmembrane protein that regulates asexual development in *A. nidulans*, and it is required for expression of *brlA* [30]. The results correlate with the different sporulation capacities presented by the mutant strains (data not shown).

The category "Transport" presented a considerable number of differentially expressed genes in both mutant strains (Table 1). In the *ΔgprB* mutant strain there were 25 transporter-encoding genes (14 up-regulated and 11 down-regulated) while in the *ΔgprD*, there were 23 transporter-encoding genes (13 up-regulated and 10 down-regulated). Many of these transporters are members of the Major Facilitator Superfamily (MFS), single-polypeptide secondary carriers able to transport only small solutes in response to chemiosmotic ion gradients [31]. Most of the genes related to MFS transporters belong to the class of multidrug resistant (MDR) transporters [32]. In the *ΔgprD* strain, a gene encoding for transport of secondary metabolites, *dbaD* (AN7898; [33]) was strongly up-regulated (Table S1). Several genes encoding amino acid transporters including AN8659, AN9300 and AN9174 were strongly down-regulated in the *ΔgprD* strain, compared to wild-type, by 3-, 4-, and 6-fold, respectively. AN8659 encodes for a gene similar to the *S. cerevisiae* gene *tat1*, which encodes for an amino acid transporter responsible for the uptake of valine, leucine, isoleucine, and tyrosine [34]–[36]. AN9300 is similar to the *S. cerevisiae* gene *MUP1*, which is involved in the synthesis of a transporter responsible for the uptake of methionine and cysteine [37], [38]. AN9174 encodes for a gene similar to the *S. cerevisiae* gene *gap1*, encoding a general amino acid permease in the yeast. GAP1 directs the uptake of all the naturally occurring L-amino acids, some D-amino acids, toxic amino acids analogs and the polyamines putrescine and spermidine [39]–[42], and appears to be transcriptionally regulated by nitrogen catabolite repression (NCR; [39]).

A previous study reported the interaction of a G-alpha subunit of the fungal pathogen *Sporothrix schenckii* with two metal ion transporters, known as siderophores [43]. The permeases are generally expressed in response to stress conditions and iron deprivation. The interaction of permeases with a component of G-protein signaling reinforces the role of G- proteins in response to environmental signals. In *A. nidulans ΔgprB* strain, we observed two highly down-regulated major iron siderophore genes, identified as AN7800, encoding the *mirA* gene and AN8540, encoding *mirB* gene [44], [45]. In *A. nidulans*, the lack of the intracellular siderophore ferricrocin contributes to several effects, including elimination of cleistothecia formation in homothallic conditions [46]. The results suggest that absence of *gprB* impairs the perception of environmental signals that lead to sexual development. Markedly, the gene *gprA* (AN2520), the homolog of *gprB* in *A. nidulans* [7], a recognized pheromone receptor, had a 3.5-fold increase in expression in the *ΔgprB* strain, compared to the wild-type strain. This finding indicates the regulation of cellular signaling is encoded by a redundant gene in the absence of its homolog.

Genes involved in interactions with the environment, cell rescue, defense, and virulence were differentially transcribed in the mutant strains. AN5046 encoding a defense-like protein, Anisin-1, annotated with high identity to the mosquito defense AaDefA1 [47] was down-regulated in both mutant strains (Supplementary Tables S1 and S2). Another gene encoding a defense-like protein, AN11510, was less expressed in the *ΔgprB* mutant strain (Supplementary Tables S1 and S2). The gene AN6487, encoding for a putative aspartyl protease similar to *S. cerevisiae yps3* gene, responsible for yapsin production involved in maintenance of cell wall integrity [48], was upregulated in the *ΔgprB* strain. Similarly, the gene identified as AN3675 had 2-fold increase in expression in the *ΔgprD* strain, when compared to the wild-type strain. This gene encodes CpcA, a transcription factor involved in cross-pathway control of amino acid biosynthesis in response to amino acid starvation, with an additional role in sexual development [49], [50].

In summary, the results obtained from the transcriptome analysis of two *A. nidulans* GPCR mutant strains, *ΔgprB* and *ΔgprD*, revealed differential expression in a variety of genes related mainly to secondary metabolism, sexual development, stress signaling, and amino acid metabolism. It appears that the absence of these GPCRs triggers a stress response at the genetic level. The data suggest an intricate relationship among different G-protein coupled receptors, fine-tune regulation of secondary and amino acid metabolisms, and fungal development.

### The metabolome of A. nidulans ΔgprB and ΔgprB strains grown on glucose

The comparison of the gene expression profiles between the *A. nidulans ΔgprB* and *ΔgprD* strains and the wild-type, grown on glucose, demonstrated that the overall metabolism of the mutant strains, especially in the *ΔgprD* strain, was strongly modified. To gain a better understanding of the metabolic processes affected by the absence of *gprB* and *gprD* genes, we analyzed the intracellular metabolites produced by the *A. nidulans* strains in the same conditions of the gene expression studies. The strains were grown on glucose in a batch cultivation medium for 24 and 48 hours at 37°C, the metabolites were extracted as described in Material and Methods, and analyzed by ^1^H NMR. Five biological replicates were used to generate the data presented below.

**Figure 6:**
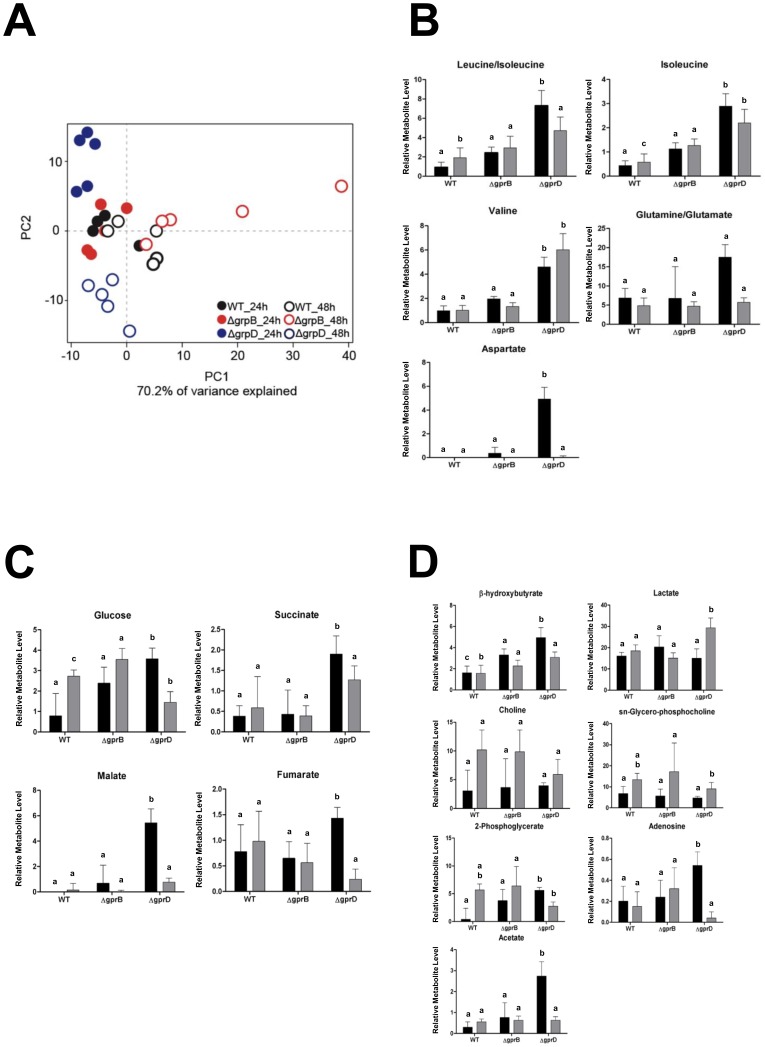
The metabolome of *A. nidulans ΔgprB* and *ΔgprB* strains grown on glucose. (A) PCA plot illustrating the variance of the integrals from NMR spectral data for all strains collected at 24 or 48 hours. (B–D) Column charts for identified metabolites expressed in Relative Metabolite Levels (Calculated taking Log2 of molar ratios from integrated spectral data). Metabolite levels were measured and averaged for each strain after 24 hours of growth (*black*) and 48 hours of growth (*light grey*). Using a two-way ANOVA, at 24 hours P<0.001 was determined for leucine/isoleucine, valine, isoleucine, β-hydroxybutyrate, acetate, malate, and aspartate, P<0.05 was determined for glucose. At 48 hours, P<0.001 was determined for valine and glucose, P<0.01 was determined for isoleucine and lactate, P<0.05 was determined for leucine/isoleucine and β-hydroxybutyrate. The columns not sharing the same letter are significantly different from strains within their time of collection (Tukey HSD, *p*<0.05).

First, the statistical method of PCA was applied to the data, which was used to qualify differences within the data set. This method reduces multidimensional data to two principal components that represent the differences among samples in a two-dimensional plot [51]. Our PCA analysis demonstrated that the principal component 1 (PC1) was responsible for 70.2% of the variance, with the PCA scores plot revealing three major groups of data points, as observed in the Figure 6A. One group is represented in the middle of the plot, and contains data points representing the wild-type and *ΔgprB* strains grown on glucose for 24 and 48 hours. The other two groups are located in the upper and lower left sides of the plot, representing the data points from the *ΔgprD* strain grown on glucose for 24 and 48 hours, respectively (Figure 6A). These results led to two main conclusions: (i) the overall intracellular metabolism of the fungi was not highly affected in the absence of *gprB* gene; (ii) there was a shift in the metabolic profile of the fungus grown on glucose in the *ΔgprD* strain. The second conclusion (ii) correlated with the previous results, which demonstrated the absence of *gprD* gene had a more perceptible effect compared to the absence of *gprB*.

In our metabolome analysis, we were able to clearly detect five different amino acids: leucine, isoleucine, valine, glutamine and aspartate (Figure 6B and Table S3). In all repetitions, the levels of these amino acids were increased in the *ΔgprD* strain, compared to the wild-type strain. The intracellular intermediates of carbon metabolism, glucose, succinate, malate, and fumarate, were detected in the present study. After 24 hours of growth on glucose, the *ΔgprD* strain presented significant differences from the wild-type and *ΔgprB* strains for all the carbon metabolism intermediates (Figure 6C and Table S3). Glucose is the most readily used carbon source by fungi, and succinate, malate and fumarate are intermediates of the tricarboxylic acid cycle (TCA).

The metabolomic analysis detected the intracellular metabolites ß-hydroxybutyrate, lactate, choline, sn-glycero-phosphocholine, 2-phosphoglycerate, adenosine, and acetate (Figure 6D). Of these metabolites, ß-hydroxybutyrate, lactate, and adenosine were significantly increased in the *ΔgprD* strain, compared to the wild-type strain, in at least one time point (Table S3). In some prokaryotes, ß-hydroxybutyrate (BHB) is the monomer of the polymeric ester poly-ß-hydroxybutyrate (PHB), an intracellular energy reserve accumulated when the microorganism is grown under nutrient limitations [52]. In mammals, BHB is one of the ketone bodies that is used as an energy source during fasting and starvation [53]. Lactate, a metabolite produced under anaerobic fermentative conditions [54] was detected at high concentrations in *A. nidulans* strains grown on glucose, suggesting that fermentation is also taking place under our experimental conditions. An accumulation of lactate indicates glycolysis is taking place at a faster rate than the TCA cycle is able to utilize all of the glycolytic end products. The levels of lactate in the *ΔgprD* strain were almost 2-fold higher at 48 hours compared to the wild-type and *ΔgprB* strains (Figure 6D).

Choline and sn-glycero-phosphocholine are important components of cellular membranes, and choline appeared to be involved in the growth and in the regulation of mycelial morphology of filamentous fungi [55]. Both metabolites were detected at relatively high levels in our experimental conditions, but no significant differences were detected among the strains (Figure 6D and Table S3).

Adenosine is a precursor of AMP, ADP, ATP, and the second messenger cAMP [56]. The levels of adenosine in the mutant *ΔgprD* were 3-fold higher compared to the wild-type strain after 24 hours of growth, but these levels strongly decreased after 48 hours (Figure 6D). The metabolite 2-phosphoglycerate is an intermediate of glycolysis. It is the substrate of the ninth step of this metabolic pathway, where it is catalyzed by enolase into phosphoenolpyruvate (PEP). 2-phosphoglycerate accumulated in a significant manner in both mutant strains after growth for 24 hours, compared to the wild-type strain (Figure 6D). Acetate is a key metabolic precursor in most organisms. In fungi, acetate is involved in many metabolic processes, from glycolysis to secondary metabolism, through generation of acetyl-CoA. Under experimental conditions, acetate was highly accumulated in the *ΔgprD* strain after 24 hours of growth, decreasing after 48 h, in a parallel manner to adenosine (Figure 6D).

In summary, the ^1^H NMR analysis of intracellular metabolism provided a rapid and efficient method to observe the metabolic fingerprint of *A. nidulans* under experimental conditions. The results clearly demonstrated the metabolome of *A. nidulans ΔgprD* strain differed significantly from the wild-type and *ΔgprB* mutant strain. The metabolic profiles of the strains reinforce the idea that the absence of a functional GprD affects glucose metabolism in *A. nidulans*. The PCA analysis demonstrated a significant metabolite shift in the *ΔgprD* strain grown on glucose (Figure 6A). A considerable number of amino acids were detected in our experimental conditions, and the amount of amino acids was higher in the *ΔgprD*, compared to the wild-type and *ΔgprB* strains (Figure 6B). This suggests that *A. nidulans* GprD could be involved somehow in the control of amino acid synthesis. In fact, recent interest in branched chain amino acids (BCAA) have shown that BCAA are major regulators via mTOR [57] and nutrient sensing [58], and induce mitochondria biogenesis [59]. In fact, they have been proposed to be potential targets for type 2 diabetes [58]. As demonstrated by the transcriptome analysis, the *cpcA* gene (AN3675) was up-regulated in the *ΔgprD* strain in the same conditions (Supplementary Table S1). This gene encodes for CpcA, a transcriptional regulator that controls the amino acid supply in fungi [49], [50], [60]. An overexpression of the *cpcA* gene induces an arrest in fruiting body formation when low levels of amino acids are present. The block can be released by a supplement of amino acids, in connection to the control of primary metabolism and fungal development [49], [50], [60]. The up-regulation of the *cpcA* transcript in *ΔgprD* may control sexual development and thereby maintain equilibrium, but an excess of amino acids releases this control. Regarding the *gprD* gene deletion, the results provide evidence for the activation of the genetic system responsible for amino acid supply and consequently fruiting bodies formation. Therefore, GprD may regulate sexual development using the aforementioned mechanism.

Asexual development is the common reproductive method in most fungi, whereas sexual reproduction is rare, due to the high energy-consumption of the sexual sporulation process [60]. The glucose metabolism detected in the *gprD* strain may not provide sufficient energy for sexual development. The ^1^H NMR analysis found metabolic intermediates, typical of starvation conditions and hypoxia, such as, ß-hydroxybutyrate and lactate, respectively. The high amount of lactate may suggest that fermentation was taking place in our experimental conditions in the presence of oxygen. It is well known in mammalian systems, that the ‘fight or flight’ response induced by epinephrine significantly increases glucose consumption in the periphery [61], but glucose production in the liver [62], It is mediated by a G-protein coupled receptor which activates PKA and increases cAMP levels [62]. A recent study has shown that a similar decrease in PKA activity with increased glucose consumption as occurred with the *ΔgprD* strain occurs with the α-adrenoreceptor (G-protein coupled receptor) in adipocytes.

The levels of acetate, malate, succinate, and fumarate were increased in the *ΔgprD* strain after growth for 24 h. In addition to the TCA cycle, succinate and malate are produced through an alternative pathway, the glyoxylate shunt, which allows cells to convert acetyl-CoA, provided from acetate, into succinate. Succinate can then be used to replenish the TCA cycle or to function as precursors for amino acid biosynthesis or carbohydrate biosynthesis. Thus, the glyoxylate shunt enables cells to use acetate as the sole carbon source [63]. This pathway may be activated in the absence of GprD, providing the amino acids and the energy necessary for fruiting bodies formation. However, the unpaired metabolism is probably a cause for the developmental defects observed in the *ΔgprD* strain [4].

### Conclusions

Transcriptional profiling and NMR analysis aimed to identify the metabolic pathways that are influenced in *A. nidulans* by GPCRs, GprB and GprD. Notably, the *A. nidulans* wild-type PKA activity is increased upon starvation. However, upon starvation, the PKA activity is increased in the *ΔgprB* mutant but decreased in the *ΔgprD* mutant strains. The *ΔgprB* and *ΔgprD* strains displayed contrasting behavior, *i.e.* the absence of GprB promoted the increase of PKA activity during growth on glucose and during starvation. Although, the absence of GprD drastically reduced the activity of PKA, the decrease was more evident under starvation conditions. Our results suggest that the absence of *gprB* and *gprD* genes interferes, at both transcriptional and metabolic levels, with the different metabolic pathways of *A. nidulans*. A large number of genes encoding proteins involved in secondary metabolism were modulated at the transcriptional level in both mutants. Several members of the sterigmatocystin-aflatoxin gene cluster presented down-regulation in both mutant strains. Many of the genes of the NR-PKS monodictyphenone biosynthesis cluster had increased and decreased mRNA accumulation in *ΔgprB* and *ΔgprD*, respectively. The differential behavior between both mutants was observed for genes related to sexual development, stress signaling, and amino acid metabolism. The ^1^H NMR analysis of intracellular metabolites showed the absence of the *gprB* gene did not significantly affect overall metabolism in *A. nidulans*. The effects of the absence of the *gprD* gene were significantly detectable in PCA and statistical analysis compared to the *ΔgprB* and wild-type strains. The metabolic profiles of the strains reinforced the idea that the absence of a functional GprD affects glucose metabolism in *A. nidulans.* Accordingly, the levels of lactate, a metabolite produced under anaerobic conditions, was 2-fold higher in the *ΔgprD* strain when compared to the wild-type and *ΔgprB* strains. The results provide a preliminary comprehension of how GprB and GprD, two fungal GPCRs, affect metabolic pathways. Further work should concentrate on understanding how GprB and GprD are able to modulate these pathways at the molecular level.

## Materials and Methods

### Fungal strains and growth conditions

The *A. nidulans* strains used in this study were kindly provided by Dr. Jae-Hyuk Yu (University of Winconsin, Madison, USA). The mutant strains RJA36 (*pabaA1*, *yA2*; *ΔgprB*::*argB* ^+^), RKH57.25 (*biA1*; *ΔgprD*::*argB* ^+^) and the wild-type strain FGSC4 (*veA* ^+^) were grown on solid minimal medium with appropriate supplements (MM), with the indicated carbon source, and incubated at 37 °C. The stock cultures were kept on silica beads with 7% milk (w/v) at 4°C. The batch cultivation medium (BCM; pH 6.5) was composed of 50 ml/liter salt solution (6 g/liter NaNO_3_, 1.5 g/liter KH_2_PO_4_, 0.5 g/liter KCl and 0.5 g/liter MgSO_4_.), 200 µl/liter trace elements (10 g/liter EDTA, 4.4 g/liter ZnSO_4_.7H_2_O, 1.0 g/liter MnCl_2_.4H_2_O, 0.32 g/liter CoCl_2_.6H_2_O, 0.315 g/liter CuSO_4_.5H_2_O, 0.22 g/liter (NH_4_)_6_Mo_7_O_24_.4H_2_O, 1.47 g/liter CaCl_2_.2H_2_O and 1 g/liter FeSO_4_.7H_2_O) and 1% glucose as carbon source. The incubation was performed at 37 °C.

For the viability assays using oxidative stress agents, 10-fold dilutions of starting suspension conidia were used. Conidia were collected from 5-day-old cultures grown on solid minimal medium containing the appropriate supplements. The amount of conidia was calculated using a counting chamber. It was spotted 10^5^, 10^4^, 10^3^ and 10^2^ conidia in a volume of 5 µl on minimal medium agar plates (control) and agar plates containing farnesol (10 and 50 µM), 2 mM paraquat, and 0.1 mM menadione. The plates were incubated at 37°C for 48 h.

For the quantification of sporulation, 50 µl of a spore suspension containing 1×10^7^ conidia prepared from a freshly harvested and filtered spore suspension was spread onto MM agar plates containing low glucose (0.1% w/v) and high glucose (1% w/v) concentration. Five plates for each strain were incubated for 48 h, and the conidia produced on each plate were harvested with 10 ml of PBS solution containing 2% (v/v) Tween 80 (Merck, Germany). The spore suspensions were filtered through 40 µm-pore filter (Millipore, USA), and the number of conidia was determined using a counting chamber.

### Construction of A. nidulans GprD::mRFP and GprB::GFP strains

To generate the GprD::mRFP and the GprB::GFP strains under the control of the endogenous promoter, a portion of 5′ UTR flanking region, the open reading frame (ORF) and a portion of 3′ UTR flanking region of the *gpr* genes were amplified from the wild-type *A. nidulans* gDNA and cloned in frame with the monomeric red fluorescent protein (*mRFP*) gene or the green fluorescent protein (GFP) gene. The stop codon of the genes was omitted in the construction. The construct links *mRFP* or *GFP* to the C-terminus of *gprD* and *gprB*, respectively, and the selective marker used was *pyro*. The *S. cerevisiae in vivo* recombination system was used for the production of the transformation cassette as previously described [64].

### Microscopic analysis

The GprD::mRFP and GprB::GFP strains were grown on coverslips in the indicated conditions. After incubation in minimal medium with no carbon source or glucose for a determined time, the coverslips were rinsed with phosphate-buffered saline (PBS; 140 mM NaCl, 2 mM KCl, 10 mM NaHPO_4_, 1.8 mM KH_2_PO_4_, pH 7.4) and mounted for examination. Slides were visualized on an Observer Z1 fluorescence microscope using a 100× objective oil immersion lens (mRFP filter; excitation 543 nm/emission 550 nm). DIC (differential interference contrast) images and fluorescence images were captured with an AxioCam camera (Carl Zeiss) and processed using AxioVision software (version 4.8).

### RNA extraction

After harvesting, mycelia were disrupted by grinding, and total RNA was extracted with RNeasy Plant Mini Kit (Qiagen). RNA (10 µg) from each treatment was fractionated in 2.2 M formaldehyde, 1.2% agarose gel, stained with ethidium bromide, and visualized with UV-light in order to check RNA integrity. The samples were submitted to RNAse-free DNAse treatment as previously described, purified with RNeasy® Mini Kit (Qiagen), and then quantified in the NanoDrop® 2000 Thermo Scientific (Thermo Scientific). RNA integrity was verified in the Agilent 2100 Bioanalyzer (Agilent Technologies), according to manufacturer's protocol.

### Protein kinase A activity assay

A total of 10^7^ conidia of the wild-type, *ΔgprB*, and *ΔgprD* mutant strains were grown for 24 hours at 37°C in erlemeyer-flasks of 250 ml 50 ml MM containing 2% glucose on a rotary shaker (180 rpm). The liquid media was discarded and the mycelia washed with sterile water prior incubation with glucose-free MM for 12 and 24 hours, under the same conditions. The mycelia was harvested via vacuum filtration and immediately frozen in liquid nitrogen. The extraction of total protein and the protein kinase A activity assays were performed using the PepTag® Non-Radioactive Protein Kinase A kit (Promega), according to the manufactures instructions. The intensity of the phosphorylated substrate was determined via densitometry analysis using the ImageJ software.

### Microarray slides construction and gene expression methods

The Agilent arrays were designed using the Agilent E-array software tool (available at https://earray.chem.agilent.com/earray/). Briefly, gene sequences representing the whole *A. nidulans* A4 genome were uploaded and the ORF numbers carefully validated by comparing the sequences deposited in three databanks [CADRE (The Central Aspergillus Resource); AspGD (Aspergillus Genome Database) and BROAD Institute], resulting in 11,251 ORFs being submitted to Agilent E-array. Based on the quality parameter implemented by Agilent E-array, 11,143 probes were designed from the uploaded sequences. These probes were represented three or four times on the microarray slides. The gene annotation used in the analysis was based on [65], [66]. Therefore, the microarray slides comprised of 45,220 features representing the gene probes, 1,417 Agilent internal controls and 800 controls that represented 80 randomly chosen *A. nidulans* ORFs.

To identify the *A. nidulans* genes transcriptionally activated by growth of wild-type, Δ*gprB* and Δ*gprD* strains on 1% glucose for 24 and 48 h, we measured gene expression using Agilent custom-designed oligonucleotides arrays (4×44K microarray), described above. After RNA isolation and purification, cDNA was synthesized using the T7 promoter primer. Initially, 5 µg of total RNA was incubated with Agilent™ RNA Spike-In controls probes (RNA Spike A and B mix). The RNA and primer were denatured at 65°C for 10 minutes, and then placed on ice for 5 min. Subsequently, cDNA Master Mix (4 µL 5× First Strand Buffer, 2 µL 0.1 M DTT, 1 µL 10 mM dNTP mix, 1 µL MMLV-RT and 0.5 µL RNaseOut) was added, prior to incubation at 40 °C for 2 hours. Finally, the samples were incubated at 65°C for 15 minutes. The samples were then labeled with Cy-3 or Cy-5-dUTP using the Two-Color Microarray-based gene expression analysis kit (Quick Amp Labeling Kit, Agilent Technologies™, USA) following the manufacturer's procedures. cRNA amplification and labeling were performed by adding to the samples the Agilent™ Transcription Master Mix (20 µL 4× Transcription Buffer, 6 µL 0.1 M DTT, 8 µL NTP mix, 6.4 µL 50% PEG, 0.5 µL RNase OUT, 0.6 µL inorganic pyrophosphatase, 0.8 µL T7 RNA Polymerase, 2.4 µL Cyanine 3-CTP to control samples, or cyanine 5-CTP to treated samples, and 15.3 µL nuclease-free water), and incubating the mixtures at 40 °C for 2 hours. The labeled cRNA was purified using RNeasy® Mini Kit (Qiagen), and then quantified in the NanoDrop® 2000 (Thermo Scientific).

Prior to hybridization, 825 ng of each labeled cRNA was incubated at 60 °C for exactly 30 minutes with Agilent™ Fragmentation Mix (11 µL 10× Blocking agent, 2.2 µL 25× fragmentation buffer, and nuclease-free water to bring the volume to 52.8 µL). The fragmentation process was stopped by adding 55 µL of 2× GE Hybridization Buffer HI-RPM. Finally, 100 µL of the sample was placed onto the microarray slide, which was mounted into the Agilent™ Microarray Hybridization Chamber. The hybridization was performed in an Agilent G2545A Hybridization Oven set at 65 °C for 17 hours. After, microarray slides were washed according to Agilent's instruction and scanned using GenePix® 4000B microarray scanner (Molecular Devices, USA).

### Gene expression analysis

Gene expression data was extracted from TIFF images generated using Agilent Feature Extraction (FE) Software, version 9.5.3.1 (Agilent Technologies, USA) and the Linear Lowess algorithm to subtract the background noise and normalized intensity values. The normalized values were uploaded into the Express Converter (version 2.1, TM4 platform available at http://www.tm4.org/utilities.html), which converts the file from the Agilent to mev (multi experiment viewer) format that is compatible with the TM4 softwares used for microarray analysis (available at http://www.tm4.org/). The mev files were uploaded into the MIDAS software (TM4 platform), where the data was averaged for the replicated probes of each gene on the array and the two biological replicates of each treatment. The generated mev files were finally analyzed using TMeV (TM4 platform, Multi Experiment Viewer, available at http://www.tigr.org/software/microarray.shtml). Differentially expressed genes were statistically identified using one-class *t* test (*p*>0.01) and further filtered for those with a mean log_2_ expression ratio over 1.0. The full dataset was deposited in the Gene Expression Omnibus (GEO) from the National Center of Biotechnology Information (NCBI) with the number GSE42553 (http://www.ncbi.nlm.nih.gov/geo/query/acc.cgi?acc=GSE42553).

### Metabolite extraction

The metabolite extraction protocol used was based in *Neurospora crassa* and *Fusarium graminearum* metabolomic studies, recently described [67]–[70]. After growth of *A. nidulans* wild-type, Δ*gprB* and Δ*gprD* on batch culture medium containing 1% glucose (w/v) for 24 and 48 h, the mycelia were filtered under vacuum and immediately frozen at −80°C. The metabolites were extracted from frozen tissues (0.1 g) by pulverizing it in liquid nitrogen until reached the consistency of a fine powder. To each powdered sample was added 800 µl of extraction buffer containing 1∶1 (v/v) acetonitrile-*d3* (CD_3_CN):deuterium oxide (D_2_O) plus 50 mM sodium acetate-d3 (CD_3_COOD). Acetonitrile has been shown to precipitate proteins without small molecule loss [64], [66]. Extracts were clarified by centrifugation at full speed for 2 min., and the supernatants transferred to a new tube and immediately frozen on dry ice for further processing to ^1^H NMR analysis.

### ^1^H NMR analysis

For NMR-based metabolomic analysis, dried media samples were resuspended in 70 µl of deuterium oxide (D_2_O) with 20 mM phosphate buffer, 0.1 mM 2,2′,3,3′ deutero-trimethyl propionate (TSP), and 1 mM Formate. Proton (^1^H) spectra were acquired at 25 °C on a 14.1 T Varian INOVA spectrometer (600 MHz ^1^H frequency) equipped with a CapNMR™ microcoil (Magnetic Resonance Microsensors Corp, Savoy, MN). The ^1^H NMR spectra were acquired using a one-pulse sequence with pre-saturation of the water resonance using a 90°flip angle, and a total repetition time (TR) of 5.65 s. The time to acquire a spectrum from each sample was approximately 40 minutes with 512 transients.

Molar ratios were calculated from the ^1^H NMR spectra by comparing peak areas to the total integration of each spectrum after removing the water and TSP peaks. The spectral data of the culture media was processed using ACDLabs 12.0 1D NMR Processor (ACD Labs). Spectral processing followed a standard routine. First, prior to Fourier transformation, the spectra were zero-filled to 32,000 points and an apodization Gaussian function of 0.5 Hz was applied. Spectrum were phased, baseline corrected, and reference to TSP set to 0 ppm. Chemical shifts presented were obtained from the Human Metabolome Database (http://www.hmdb.ca). For statistical comparison of multiple spectra, we performed principal component analysis (PCA) [70]. The spectra were binned into 0.04 ppm segments using ACDLabs, and values were exported to SIMCA-P+11 (Umetrics). The first two principal components were plotted to demonstrate the separation between samples, which qualified differences within the dataset.

## Supporting Information

Table S1 **Differentially expressed genes in** ***ΔgprD*** **strain grown on glucose in a significant manner (p<0.01).**
(XLSX)

Table S2 **FunCat categorization of some genes down-or up-regulated in the mutant strains grown on glucose, compared to the WT.**
(XLSX)

Table S3 **Statistical analysis of metabolite profile.**
(XLSX)

## References

[1] Neves SR, Ram PT, Iyengar R (2002) G protein pathways. Science 296: 1636–1639. 12040175 10.1126/science.1071550

[2] Bahn YS, Xue C, Idnurm A, Rutherford JC, Heitman J, et al (2007) Sensing the environment: lessons from fungi. Nat Rev Microbiol 5: 57–69. 17170747 10.1038/nrmicro1578

[3] Lengeler KB, Davidson RC, D'souza C, Harashima T, Shen WC, et al (2000) Signal transduction cascades regulating fungal development and virulence. Microbiol Mol Biol Rev 64: 746–785. 11104818 10.1128/mmbr.64.4.746-785.2000 PMC99013

[4] Han KH, Seo JA, Yu JH (2004) A putative G protein-coupled receptor negatively controls sexual development in *Aspergillus nidulans* . Mol Microbiol 51: 1333–1345. 14982628 10.1111/j.1365-2958.2003.03940.x

[5] Lafon A, Han KH, Seo JA, Yu JH, d'Enfert C (2006) G-protein and cAMP-mediated signaling in aspergilli: A genomic perspective. Fungal Genet Biol 43: 490–502. 16546420 10.1016/j.fgb.2006.02.001

[6] Yu JH (2010) Regulation of development in *Aspergillus nidulans* and *Aspergillus fumigatus* . Mycobiology 38: 229–237. 23956662 10.4489/MYCO.2010.38.4.229 PMC3741515

[7] Seo JA, Han KH, Yu JH (2004) The *gprA* and *gprB* genes encode putative G protein- coupled receptors required for self-fertilization in *Aspergillus nidulans* . Mol Microbiol 53: 1611–1623. 15341643 10.1111/j.1365-2958.2004.04232.x

[8] Affeldt KJ, Brodhagen M, Keller NP (2012) *Aspergillus* oxylipin signaling and quorum sensing pathways depend on G protein-coupled receptors. Toxins 4: 695–717. 23105976 10.3390/toxins4090695 PMC3475224

[9] Gehrke A, Heinekamp T, Jacobsen ID, Brakhage AA (2010) Heptahelical receptors GprC and GprD of *Aspergillus fumigatus* are essential regulators of colony growth, hyphal morphogenesis, and virulence. Appl Environ Microb 76: 3989–3998. 10.1128/AEM.00052-10 PMC2893470 20418440

[10] Luttrell LM (2008) Reviews in molecular biology and biotechnology: transmembrane signaling by G protein-coupled receptors. Mol Biotechnol 39: 239–264. 18240029 10.1007/s12033-008-9031-1

[11] Dechant R, Peter M (2008) Nutrient signals driving cell growth. Curr Opin Cell Biol 20: 678–87. 18930818 10.1016/j.ceb.2008.09.009

[12] Zaman S, Lippman SI, Zhao X, Broach JR (2008) How *Saccharomyces* responds to nutrients. Annu Rev Genet 42: 27–81. 18303986 10.1146/annurev.genet.41.110306.130206

[13] Nakafuku M, Obara T, Kaibuchi K, Miyajima I, Miyajima A, et al (1988) Isolation of a second yeast *Saccharomyces cerevisiae* gene (GPA2) coding for guanine nucleotide-binding regulatory protein: studies on its structure and possible functions. Proc Natl Acad Sci USA 85: 1374–78. 2830616 10.1073/pnas.85.5.1374 PMC279773

[14] Rolland F, De Winde JH, Lemaire K, Boles E, Thevelein JM, et al (2000) Glucose-induced cAMP signalling in yeast requires both a G-protein coupled receptor system for extracellular glucose detection and a separable hexose kinase-dependent sensing process. Mol Microbiol 38: 348–358. 11069660 10.1046/j.1365-2958.2000.02125.x

[15] Vandamme J, Castermans D, Thevelein JM (2012) Molecular mechanisms of feedback inhibition of protein kinase A on intracellular cAMP accumulation. Cell Signal 24: 1610–1618. 22522182 10.1016/j.cellsig.2012.04.001

[16] Thevelein JM, Geladé R, Holsbeeks I, Lagatie O, Popova Y, et al (2005) Nutrient sensing systems for rapid activation of the protein kinase A pathway in yeast. Biochem Soc Trans 33(Pt 1): 253–256. 10.1042/BST0330253 15667319

[17] von Düren H (2009) A survey of nonribosomal peptide synthetase (NRPS) genes in *Aspergillus nidulans* . Fungal Genetics and Biology 46: S45–S52. 18804170 10.1016/j.fgb.2008.08.008

[18] Hynes MJ, Murray SL (2010) ATP-citrate lyase is required for production of cytosolic acetyl coenzyme A and development in *Aspergillus nidulans* . Eukaryot Cell 9: 1039–1048. 20495057 10.1128/EC.00080-10 PMC2901662

[19] Son H, Lee J, Park AR, Lee YW (2011) ATP citrate lyase is required for normal sexual and asexual development in *Gibberella zeae* . Fungal Genet Biol 48: 408–417. 21237280 10.1016/j.fgb.2011.01.002

[20] Hicks J, Lockington RA, Strauss J, Dieringer D, Kubicek CP, et al (1997) RcoA has pleiotropic effects on *Aspergillus nidulans* cellular development. Mol Microbiol 39: 1482–1493. 10.1046/j.1365-2958.2001.02332.x 11260466

[21] Calvo AM, Wilson RA, Bok JW, Keller NP (2002) Relationship between secondary metabolism and fungal development. Microbiol Mol Biol Rev 66: 447–459. 12208999 10.1128/MMBR.66.3.447-459.2002 PMC120793

[22] Roze LV, Beaudry RM, Keller NP, Linz JE (2004a) Regulation of aflatoxin synthesis by FadA/cAMP/protein kinase A signaling in *Aspergillus parasiticus* . Mycopathologia 158: 219–232. 15518351 10.1023/b:myco.0000041841.71648.6e

[23] Shimizu K, Keller N (2001) Genetic involvement of a cAMP-dependent protein kinase in a G protein signaling pathway regulating morphological and chemical transitions in *Aspergillus nidulans* . Genetics 157: 591–600. 11156981 10.1093/genetics/157.2.591 PMC1461531

[24] Shimizu K, Hicks JK, Huang TP, Keller NP (2003) Pka, Ras and RGS protein interactions regulate activity of AflR, a Zn(II)2Cys6 transcription factor in *Aspergillus nidulans* . Genetics 165: 1095–1104. 14668367 10.1093/genetics/165.3.1095 PMC1462812

[25] Roze LV, Chanda A, Linz JE (2011) Compartmentalization and molecular traffic in secondary metabolism: a new understanding of established cellular processes. Fungal Genet Biol 48: 35–48. 20519149 10.1016/j.fgb.2010.05.006 PMC2949687

[26] Sanchez JF, Somoza AD, Keller NP, Wang CC (2012) Advances in *Aspergillus* secondary metabolite research in the post-genomic era. Nat Prod Rep 29: 351–371. 22228366 10.1039/c2np00084a PMC4568942

[27] Briza P, Eckerstorfer M, Breitenbach M (1994) The sporulation-specific enzymes encoded by the DIT1 and DIT2 genes catalyze a two-step reaction leading to a soluble LL-dityrosine-containing precursor of the yeast spore wall. Proc Natl Acad Sci USA 91: 4524–4528. 8183942 10.1073/pnas.91.10.4524 PMC43818

[28] Stringer MA, Dean RA, Sewall TC, Timberlake WE (1991) Rodletless, a new *Aspergillus* developmental mutant induced by directed gene inactivation. Genes Dev 5: 1161–1171. 2065971 10.1101/gad.5.7.1161

[29] Chang YC, Timberlake WE (1993) Identification of *Aspergillus brlA* response elements (BREs) by genetic selection in yeast. Genetics 133: 29–38. 8417986 10.1093/genetics/133.1.29 PMC1205295

[30] Soidi-Raggi G, Sánchez O, Aguirre J (2006) TmpA, a member of a novel family of putative membrane flavoproteins, regulates asexual development in *Aspergillus nidulans* . Mol Microbiol 59: 854–869. 16420356 10.1111/j.1365-2958.2005.04996.x

[31] Pao SS, Paulsen IT, Saier MH Jr (1998) The major facilitator superfamily. Microbiol Molec Biol Rev 62: 1–32. 9529885 10.1128/mmbr.62.1.1-34.1998 PMC98904

[32] Sá-Correia I, Tenreiro S (2002) The multidrug resistance transporters of the major facilitator superfamily, 6 years after the disclosure of *Saccharomyces cerevisiae* genome sequence. J Biotechnol 98: 215–226. 12141988 10.1016/s0168-1656(02)00133-5

[33] Gerke J, Bayram O, Feussner K, Landesfeind M, Shelest E, et al (2012) Breaking the silence: protein stabilization uncovers silenced biosynthetic gene clusters in the fungus *Aspergillus nidulans* . Appl Environ Microbiol 78: 8234–8244. 23001671 10.1128/AEM.01808-12 PMC3497355

[34] Schimidt A, Hall MN, Koller A (1994) Two FK506 resistance-conferring genes in *Saccharomyces cerevisiae*, TAT1 and TAT2, encode amino acid permeases mediating tyrosine and tryptophan uptake. Mol Cell Biol 14: 6597–6606. 7523855 10.1128/mcb.14.10.6597-6606.1994 PMC359189

[35] Bajmoczi M, Sneve M, Eide DJ, Drewes LR (1998) TAT1 encodes a low-affinity histidine transporter in *Saccharomyces cerevisiae* . Biochem Biophys Res Commun 243: 205–209. 9473505 10.1006/bbrc.1998.8082

[36] Regenberg B, Düring-Olsen L, Kielland-Brandt MC, Holmberg S (1999) Substrate specificity and gene expression of the amino-acid permeases in *Saccharomyces cerevisiae* . Curr Genet 36: 317–328. 10654085 10.1007/s002940050506

[37] Isnard AD, Thomas D, Surdin-Kerjan Y (1996) The study of methionine uptake in *Saccharomyces cerevisiae* reveals a new family amino acids permease. J Mol Biol 262: 473–484. 8893857 10.1006/jmbi.1996.0529

[38] Kosugi A, Koizumi Y, Yanagida F, Udaka S (2001) MUP1, high affinity methionine permease, is involved in cysteine uptake by *Saccharomyces cerevisiae* . Biosci Biotechnol Biochem 65: 728–731. 11330701 10.1271/bbb.65.728

[39] Jauniaux JC, Grenson M (1990) GAP1, the general amino acid permease gene of *Saccharomyces cerevisiae*. Nucleotide sequence, protein similarity with the other bakers yeast amino acid permeases, and nitrogen catabolite repression. Eur J Biochem 190: 39–44. 2194797 10.1111/j.1432-1033.1990.tb15542.x

[40] Stanbrough M, Magasanik B (1995) Transcriptional and posttranslational regulation of the general amino acid permease of *Saccharomyces cerevisiae* . J Bacteriol 177: 94–102. 7798155 10.1128/jb.177.1.94-102.1995 PMC176561

[41] Regenberg B, Hansen J (2000) GAP1, a novel selection and counter selection marker for multiple gene disruptions in *Saccharomyces cerevisiae* . Yeast 16: 1111–1119. 10953083 10.1002/1097-0061(20000915)16:12<1111::AID-YEA611>3.0.CO;2-3

[42] Uemura T, Kashiwagi K, Igarashi K (2005) Uptake of putrescine and spermidine by Gap1p on the plasma membrane of *Saccharomyces cerevisiae* . Biochem Biophys Res Commun 328: 1028–1033. 15707981 10.1016/j.bbrc.2005.01.064

[43] Pérez-Sánchez L, González E, Colón-Lorenzo E, González-Velázquez W, González-Méndez R, et al (2010) Interaction of the heterotrimeric G protein alpha subunit SSG-1 of *Sporothrix schenckii* with proteins related to stress response and fungal pathogenicity using a yeast two-hybrid assay. BMC Microbiol 10: 317. 21143936 10.1186/1471-2180-10-317 PMC3018405

[44] Oberegger H, Schoeser M, Zadra I, Abt B, Haas H (2001) SREA is involved in regulation of siderophore bosynthesis, utilization and uptake in *Aspergillus nidulans* . Mol Microbiol 41: 1077–1089. 11555288 10.1046/j.1365-2958.2001.02586.x

[45] Haas H, Schoeser M, Lesuisse E, Ernst JF, Parson W, et al (2003) Characterization of the *Aspergillus nidulans* transporters for the siderophores enterobactin and triacetylfusarinine C. Biochem J. 371: 505–513. 10.1042/BJ20021685 PMC1223275 12487628

[46] Eisendle M, Schrettl M, Kragl C, Müller D, Illmer P, et al (2006) The intracellular siderophore ferricrocin is involved in iron storage, oxidative-stress resistance, germination, and sexual development in *Aspergillus nidulans* . Eukaryot Cell 5: 1596–603. 17030991 10.1128/EC.00057-06 PMC1595343

[47] Eigentler A, Pócsi I, Marx F (2012) The anisin1 gene encodes a defensin-like protein and supports the fitness of *Aspergillus nidulans* . Arch Microbiol 194: 427–437. 22113351 10.1007/s00203-011-0773-y PMC3354322

[48] Krysan DJ, Ting EL, Abeijon C, Kroos L, Fuller RS (2005) Yapsins are a family of aspartyl proteases required for cell wall integrity in *Saccharomyces cerevisiae* . Eukariot Cell 4: 1364–1374. 10.1128/EC.4.8.1364-1374.2005 PMC1214537 16087741

[49] Hoffmann B, Wanke C, Lapaglia SK, Braus GH (2000) c-Jun and RACK1 homologues regulate a control point for sexual development is *Aspergillus nidulans* . Mol Microbiol 37: 28–41. 10931303 10.1046/j.1365-2958.2000.01954.x

[50] Hoffmann B, Valerius O, Andermann M, Braus GH (2001) Transcriptional autoregulation and inhibition of mRNA translation of amino acid regulator gene cpcA of filamentous fungus *Aspergillus nidulans* . Mol Biol Cell 12: 2846–2857. 11553722 10.1091/mbc.12.9.2846 PMC59718

[51] Ringner M (2008) What is principal component analysis? Nat Biotechnol 26: 303–304. 18327243 10.1038/nbt0308-303

[52] Peoples OP, Sinskey AJ (1989) Poly-ß-hydroxybutyrate (PHB) biosynthesis in *Alcaligenes eutrophus* H16. Identification and characterization of the PHB polymerase gene (phbC). J Biol Chem 264: 15298–15303. 2670936

[53] Veech RL, Chance B, Kashiwaya Y, Lardy HA, Cahill GF Jr (2001) Ketone bodies, potential therapeutic uses. IUBMB Life 51(4): 241–247. 11569918 10.1080/152165401753311780

[54] Barker BM, Kroll K, Vodisch M, Mazurie A, Kniemeyer O, et al (2012) Transcriptomic and proteomic analyses of the *Aspergillus fumigatus* hypoxia response using an oxygen-controlled fermenter. BMC Genomics 13: 62. 22309491 10.1186/1471-2164-13-62 PMC3293747

[55] Markham P, Robson GD, Bainbridge BW, Trinci AP (1993) Choline: its role in the growth of filamentous fungi and the regulation of mycelial morphology. FEMS Microbiol Rev 10: 287–300. 8318261 10.1111/j.1574-6968.1993.tb05872.x

[56] Pall ML (1981) Adenosine 3′-5′-phosphate in fungi. Microbiol Rev 45: 462–480. 6272081 10.1128/mr.45.3.462-480.1981 PMC281520

[57] Dodd KM, Tee AR (2012) Leucine and mTORC1: a complex relationship. Am J Physiol Endocrinol Metab 302: E1329–E1342. 22354780 10.1152/ajpendo.00525.2011

[58] Yoshizawa F (2012) New therapeutic strategy for amino acid medicine: notable functions of branched chain amino acids as biological regulators. J Pharmacol Sci 118: 149–155. 22293293 10.1254/jphs.11r05fm

[59] Valerio A, D'Antona G, Nisoli E (2011) Branched-chain amino acids, mitochondrial biogenesis, and healthspan: an evolutionary perspective. Aging 3: 464–478. 21566257 10.18632/aging.100322 PMC3156598

[60] Bayram O, Braus GH (2012) Coordination of secondary metabolism and development in fungi: the velvet family of regulatory proteins. FEMS Microbiol Rev 36: 1–24. 21658084 10.1111/j.1574-6976.2011.00285.x

[61] Cahova M, Palenickova E, Papackova Z, Dankova H, Skop V, et al (2012) Epinephrine-dependent control of glucose metabolism in white adipose tissue: the role of α- and β-adrenergic signalling. Exp Biol Med 237: 211–218. 10.1258/ebm.2011.011189 22302710

[62] Tikunov AP, Winnike JH, Tech K, Jeffries RE, McClelland RE, et al (2013) Fluxomics by NMR Spectroscopy from Cells to Organisms Focusing on Liver. Current Metabolomics 1: 1–32.

[63] Kunze M, Pracharoenwattana I, Smith SM, Hartig A (2006) A central role for the peroxisomal membrane in glyoxylate cycle function. Biochim Biophys Acta 1763: 1441–1452. 17055076 10.1016/j.bbamcr.2006.09.009

[64] Dinamarco TM, Freitas FZ, Almeida RS, Brown NA, dos Reis TF, et al (2012) Functional characterization of an *Aspergillus fumigatus* calcium transporter (PmcA) that is essential for fungal infection. PLoS ONE 7: e37591. 22649543 10.1371/journal.pone.0037591 PMC3359301

[65] Arnaud MB, Chibucos MC, Costanzo MC, Crabtree J, Inglis DO, et al (2010) The Aspergillus genome database, a curated comparative genomics resource for gene, protein and sequence information for the *Aspergillus* research community. Nucleic Acids Res 38: D420–D427. 19773420 10.1093/nar/gkp751 PMC2808984

[66] Galagan JE, Calvo SE, Cuomo C, Ma LJ, Wortman JR, et al (2005) Sequencing of *Aspergillus nidulans* and comparative analysis with *A. fumigatus* and *A. oryzae* . Nature 438: 1105–1115. 16372000 10.1038/nature04341

[67] Kim JD, Kaiser K, Larive CK, Borkovich KA (2011) Use of 1H NMR to measure intracellular metabolite levels during growth and asexual sporulation in *Neurospora crassa* . Eukaryot Cell 10: 820–831. 21460191 10.1128/EC.00231-10 PMC3127669

[68] Lowe RG, Allwood JW, Galster AM, Urban M, Daudi A, et al (2010) A combined (1)H nuclear magnetic resonance and electrospray ionization-mass spectrometry analysis to understand the basal metabolism of plant-pathogenic *Fusarium* spp. Mol Plant Microbe Interact 23: 1605–1618. 20718668 10.1094/MPMI-04-10-0092

[69] Boernsen KO, Gatzek S, Imbert G (2005) Controlled protein precipitation in combination with chip-based nanospray infusion mass spectrometry. An approach for metabolomics profiling of plasma. Anal Chem 77: 7255–7264. 16285673 10.1021/ac0508604

[70] Stacklies W, Redestig H, Scholz M, Walther D, Selbig J (2007) pcaMethods a bioconductor package providing PCA methods for incomplete data. Bioinformatics 23: 1164–1167. 17344241 10.1093/bioinformatics/btm069

